# Novel synthesis of Ni/Fe layered double hydroxides using urea and glycerol and their enhanced adsorption behavior for Cr(VI) removal

**DOI:** 10.1038/s41598-020-57519-4

**Published:** 2020-01-17

**Authors:** Gehad Y. Abo El-Reesh, Ahmed A. Farghali, Mohamed Taha, Rehab K. Mahmoud

**Affiliations:** 1 Chemistry Department, Faculty of Science, Beni-Suef University, Beni-Suef, Egypt; 2 Materials Science and nanotechnology Department, Faculty of Postgraduate Studies for Advanced Sciences (PSAS), Beni-Suef University, Beni-Suef, Egypt

**Keywords:** Environmental sciences, Chemistry, Materials science

## Abstract

Novel modified Ni/Fe layered double hydroxides with different morphology of spherical – like shape were fabricated via using urea as a ligand and glycerol (Ni/Fe LDH/GL) with Ni:Fe molar ratios of 2:1 by the simplest co -precipitation method. Also, for comparison purposes, Ni/Fe LDH was synthesized to be used as a control one. A suggested interpretation for the morphology change was also given. The materials were characterized by X-ray diffraction (XRD), The Fourier transform infrared (FT - IR) spectroscopy, field emission scanning electron microscopy (FESEM), EDX for elemental analysis, high resolution transmission electron microscopy (HRTEM), Brunauer, Emmett, and Teller (BET) equation, particle size distributions and Zeta potential measurements. In addition, the synthesized materials were used as adsorbents for removal of potassium dichromate from aqueous solutions under various experimental conditions. The adsorption of Cr (VI) was strongly pH dependant and the pH_PZC_ was studied. Kinetic studies were evaluated through different models including, pseudo first and second orders, mixed 1, 2 orders, intra particle diffusion and Avrami models. For adsorption isotherms, two-parameter models (Langmuir, Freundlich and Temkin) and three parameter models (Sips, Langmuir-Freundlich and Tooth) were investigated showing maximum adsorption capacity of 50.43 mg/g and 136.05 mg/g for Ni/Fe LDH and Ni/Fe LDH/GL, respectively. Also, the effect of temperature was investigated at (23, 35, 45, 55 °C) and the thermodynamic parameters (∆H°, ∆S° and ∆G°) were calculated showing exothermic and spontaneous adsorption process. The effect of coexisting anions (Cl^−^, SO_4_^2−^ and HPO_4_^2−^) and humic acid at different concentrations on the removal efficiency of dichromate ions was investigated. Chemical stability and recyclability of these adsorbents were also studied. The intermolecular hydrogen bonds formation between dichromate ion, urea, glycerol, LDH was explored by Monte Carlo simulation This study suggested that the modified Ni/Fe LDH/GL materials were promising nanoadsorbents for efficient potassium dichromate removal.

## Introduction

Water is the most precious natural source important for human beings, agriculture, and industrial activities. Recently, water bodies are potentially contaminated and polluted by many hazardous materials such as nuclear and industrial wastes, pharmaceuticals, organic contaminants and heavy metals. Heavy metals like cadmium, zinc, copper, lead, arsenic, mercury and chromium have significant threat to human health due to its potentially toxic effects^1,2^. Chromium is one of heavy metals widely used in many industrial processes such as electroplating, dyes, mining, photography, textile and leather^3^. Chromium present in water in a trivalent and hexavalent oxidation state, it is established that hexavalent chromium exhibits high toxicity toward living organisms than trivalent chromium which in contrast is a micronutrient^4^. Hexavalent chromium is reported as a cause of respiratory cancers^5,6^ and mutations and chromosomal damage^7^. Carcinogenicity of drinking water containing dichromate in oral cavity and small intestine has been evaluated in the National Toxicology Program (NTP) recent reports^8^ chromate dose of 0.10 mg/l in water is the acceptable limit recommended by U.S. EPA^9^. Due to its potential toxicological effects, it has been become necessary and urgent to remove chromium from drinking water. Many treatment techniques such as ion exchange, chemical precipitation, reverse osmosis, membrane separation, solvent extraction and filtration^10–14^ have been suggested for removal and up take of dichromate from water. Adsorption is considered as an effective, economical and environmentally friendly method for removal of heavy metal ions from aqueous solutions and attracts significant attention in the treatment of water^15^. Recently, many adsorbents such as activated carbons^16^, polymeric materials^17–19^, graphene based materials^20,21^, magnetic materials^22^, modified clays^23,24^, hybrid materials^19,25^, and biomaterials^26^ have been obeyed for wastewater treatment through adsorption process. Many efforts have been devoted to the removal of pollutants from water and various sorbents have been applied and developed in the industrial wastewater treatment^27–31^. However, in spite of the advantages of these sorbents like production cost over efficiency, wastewater treatment methods should be still improved to minimize costs by reducing reaction time or recycling the materials. In addition, these sorbents suffer from complicated synthesis, the relatively low adsorption capacity, tendency to agglomeration like TiO_2_^32^, needing high cost for modification and also removing of one only pollutant type which have high impact on the removal efficiency^33^. So, the improvements of efficiency and reuse of catalysts has been one of the main interests and the need for developing new novel and multifunctional adsorbents for highly efficient adsorption become important and urgent.

Layered double hydroxides (LDHs) are 2D structured inorganic layered compounds with chemical structure represented by the formula [M_1−x_^2+^M_x_^3+^(OH)_2_]^x+^(A^n−^)_x/n_·mH_2_O, where, M^+2^ is a divalent metal cation (such as Zn^2+^, Ni^2+^, Co^2+^ and Mg^2+^), M^+3^ is a trivalent metal cation (such as Fe^3+^, Al^3+^, Cr^3+^), A^n−^represents active anions (e.g., CO_3_^2−^, Cl^−^, NO_3_^−^, SO_4_^2−^) in the interlayer region and x is the molar ratio of M^2+^/(M^2+^ + M^3+^). The potential of layered double hydroxides (anionic clays) as promising adsorbent materials recognized mainly in water remediation applications to their interlayer anion exchange capacity, high specific surface area, low cost, high tunable interior structure and low toxicity properties^34–36^. The combination of Layered double hydroxides with other organic or inorganic materials in composite causes enhancement in adsorption capacity and the surface properties to be suitable for the removal process^34^. LDHs are regarded as a valuable adsorbent for removal of heavy metals and wastewater treatment arising from their unique properties including their high stability and other physicochemical properties^37^. LDHs were widely used in the removal of Cr(VI) ions from solutions as reported in many studies^38,39^ and, recently, they are used in Cr(VI) soil remediation^40^.

In spite of, the potential efficiency of LDHs in remediation of anionic waste by ion exchange reactions, they suffer from severe limitations, such as bulk mass loss during anion exchange reactions^41,42^ and some of the prepared LDH have significant solubility in water^43^. Therefore, some of the LDHs are sources of heavy metals pollutants. This severely shows restriction the choice of LDHs to those containing metal ions such as Mg, Ca, Al, and Fe metal ions. So, we have chosen the Ni/Fe LDH as a model, as it has a low solubility product (p*K*sp = 60.81) compared to other prepared LDHs^44^.

In this study, spherical-like modified Ni/Fe LDH was fabricated through introducing glycerol during LDH synthesis to obtain more desired structure characters and increase its adsorption efficiency toward hexavalent chromium ions in wastewater.

The purposes of this study are (1) to synthesize layered Ni/Fe LDH and spherical - like modified Ni/Fe LDH/GL by the simplest co-precipitation method; (2) characterize the as prepared LDH materials by using various tools such as X-ray diffraction (XRD), Fourier transform infrared spectroscopy (FT-IR), Field emission scanning electron microscopy (FESEM), High resolution transmission electron microscopy (HRTEM), Brunauer, Emmett, and Teller equation (BET), particle size distribution and Zeta potential measurements; (3) study and compare adsorption behavior of Cr(VI) ions on both Ni/Fe LDH and modified Ni/Fe LDH/GL through applying many conditions (e.g., solution pH, catalyst dose, contact time, concentration of Cr(VI) and temperature); (4) study stability and recyclability of LDH materials; (5) suggest mechanism of interaction between Cr(VI) and modified LDH materials.

## Results and Discussions

### Characterization of Ni/Fe LDH and Ni/Fe LDH/GL Nanocomposite

#### Crystal structure

**Figure 1:**
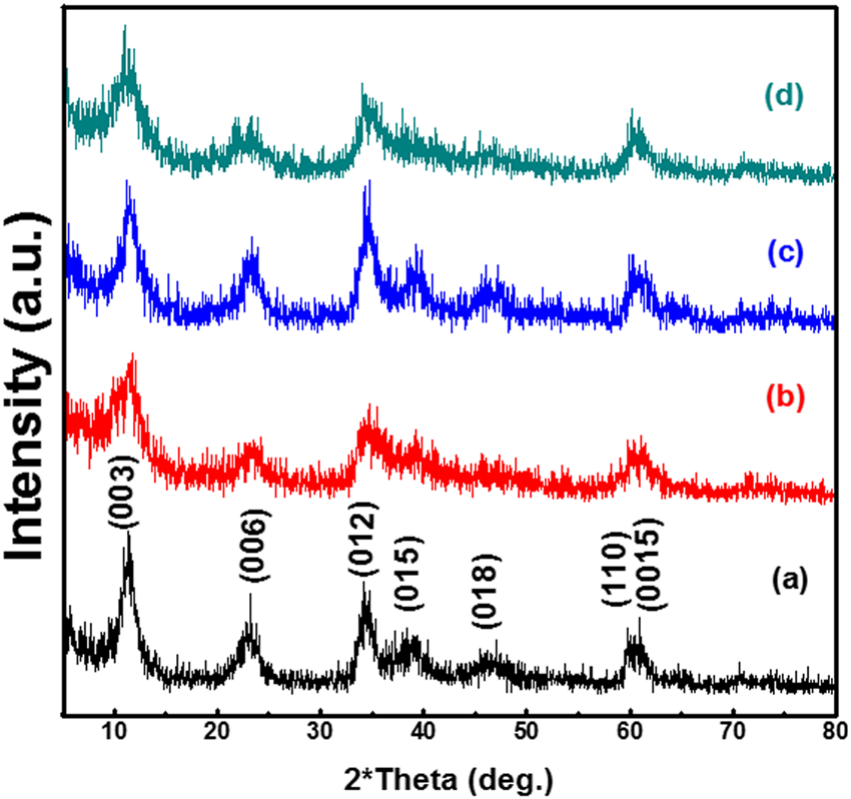
XRD diffractograms of the (**a**) Ni/Fe LDH, (**b**) Ni/Fe LDH/GL, (**c**) Ni/Fe LDH after adsorption, and (**d**) Ni/Fe LDH/GL after adsorption.

Figure 1(a,b) displays the powder X-ray patterns of the prepared Ni/Fe LDH and Ni/Fe LDH/GL Nanocomposite. Both samples show reflection peaks typical of the layered double hydroxide structure. The diffraction peaks matched those in the reported in the reference data (ICDD card no. 01-082-8040). The reflections peaks at 11.6°, 23.2°, 34.5°, 39.1°, 46.5°, 59.9°, and 60.9° could be indexed to the plane families (003), (006), (012), (015), (018), (110), and (0015) respectively. The samples possessed a Rhombohedral crystal system with R-3 space group (group number 148). The (003) interlayer distance was calculated to be (7.82 Å) for the Ni/Fe LDH which is close to the literature value^37,45^. After Glycerol loading the (003) interlayer distance did not show any significant change suggesting that the Glycerol was adsorbed on the surface of the LDH layers rather than being intercalated between the layers.

The crystallite size for Ni/Fe LDH and Ni/Fe LDH/GL was calculated to be 10.92 nm and 12.75 nm respectively and this confirm the loading of glycerol. As shown in Fig. 1(c,d), after adsorption the samples still show the same characteristic diffraction peaks compared to before adsorption and this illustrates the stability of the Ni/Fe LDH and Ni/Fe LDH/GL adsorbents used after the adsorption tests. Figure S1 represents the XRD of Ni/Fe LDH prepared by urea and NaOH.

**Figure 2:**
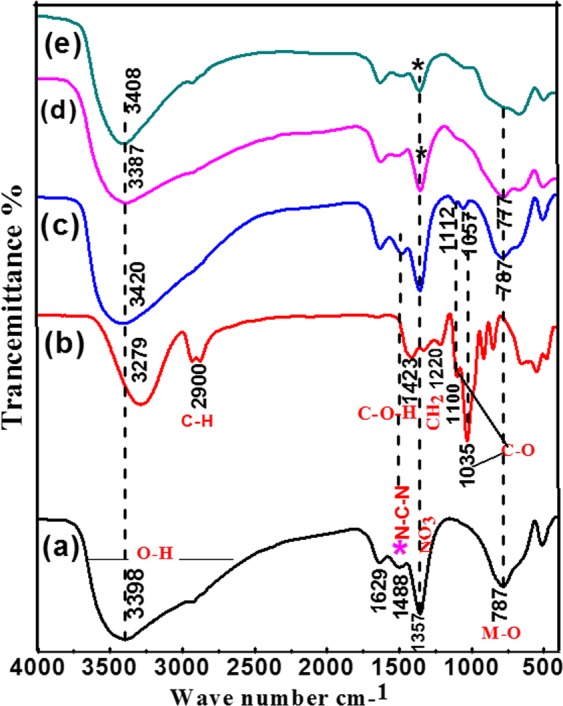
FTIR spectra of (**a**) Ni/Fe LDH, (**b**) Glycerol, (**c**) Ni/Fe LDH/GL, (**d**) Ni/Fe LDH after adsorption, and (**e**) Ni/Fe LDH/GL after adsorption.

The FT-IR spectra of the Ni/Fe LDH, Glycerol and Ni/Fe LDH/GL Nanocomposite are shown in Fig. 2(a–c) The chemical bonds in the Ni/Fe-NO_3_ LDH were identified by the band that is pointed at 787 cm^−1^ and attributed to M–O–M vibration^46,47^. This band like the M–O–H bending^47^, involved the oxygen metal ions translational motion in the brucite -like layers^48^. The strong broad band at 3398 cm^−1^ was related to the stretching vibrations of the H-bond of the OH group (ν O–H) in the brucite-like layers^49,50^. The bending vibration (δ H_2_O) of the H_2_O molecules in the interlayers^49,51^ appeared at 1629 cm^−1^. The peak located at 1357 cm^−1^ is related to the ν stretching vibration of the NO_3_ groups in the LDH interlayer. As For glycerol, The peak at 3279 cm^−1^ can be attributed to the O–H bond stretching while the bimodal peak around 2900 cm^−1^ is due to the C–H stretching^52–54^. The peak at 1423 cm^−1^ is due to the bending of C–O–H group. The peak at 1035 cm^−1^ and the other peaks in the low wave number region (1100–900 cm^−1^) are attributed to C–O stretching^52–54^ while that at 1220 cm^−1^ is due to CH2 bending^54^. As Shown in Fig. 2(c) it is interesting to note the presence of important bands of GL in the spectra of Ni/Fe LDH/GL Nano Composites indicated a successful loading of Gl in the Ni/Fe LDH host^55,56^. Besides Also, the intensity of some peak at 1357 cm^−1^ was decreased indicating the exchange of nitrate anions by the GL^56^. The main absorption peak at 3398 cm^−1^ in Ni/Fe LDH has shifted to higher wavelength 3420 cm^−1^ in Ni/Fe LDH/GL indicating the promotion of hydrogen bonding interactions between Ni/Fe LDH and glycerol^53^. The overlapping of peak at 2900 cm^−1^ with the peak at 3398 cm^−1^ and the presence of the two absorption peaks at 1112 and 1057 cm^−1^ (Fig. 2) in the Ni/Fe LDH/GL nanocomposite related to C–O stretching indicates the successful loading of the glycerol onto the LDH phase.

#### Morphology of Ni/Fe LDH and Ni/Fe LDH/GL

**Figure 3:**
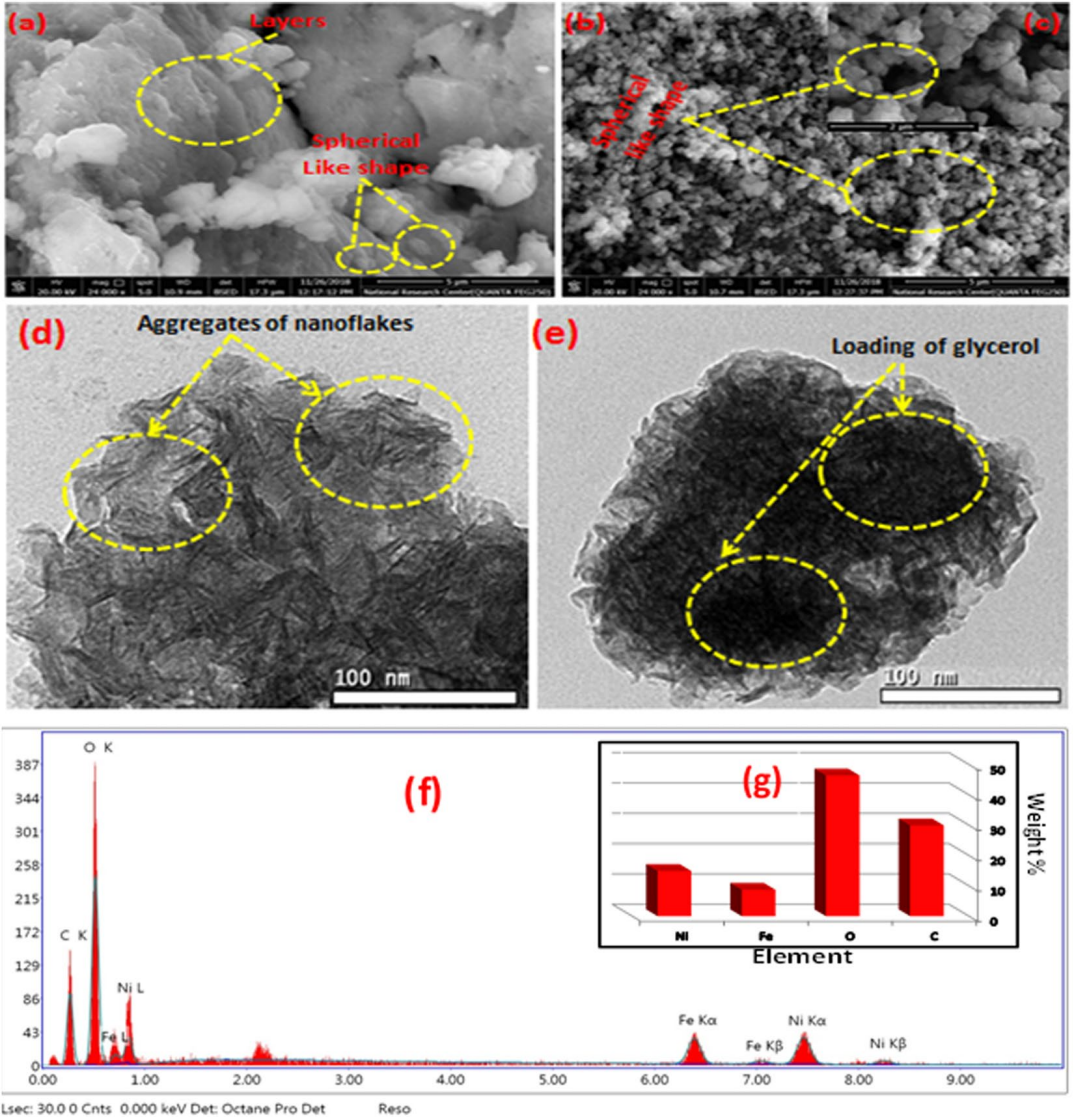
SEM images [(**a**) Ni/Fe LDH, (**b**) Ni/Fe LDH/GL and (**c**) inset high magnification], TEM images [(**d**) Ni/Fe LDH, (**e**) Ni/Fe LDH/GL] and EDX of Ni/Fe LDH/GL [(**f**) elemental composition and (**g**) quantitative percent for elements].

The morphologies of the synthesized materials were mainly affected by the experimental conditions, such as the ratio of H_2_O to GL, amount of urea used, ratio of Ni/Fe and the reaction temperature^57^. Firstly, the effect of the GL on the morphology of the prepared materials was shown in Fig. 3. It is clearly appear that without the addition of GL (Fig. 3(a)), two kinds of morphology, sphere-like and plate-like structure can be obtained. The formation of Ni/Fe-LDH microsphere was mainly through the using of urea which forming binary complex with Ni and Fe metal ions^58^ which confirmed by the appearance of a peak at 1488 cm^−1^ and this peak is related to N–C–N of urea^59^ as shown in Fig. S2 which represents the FTIR of Ni/Fe LDHs prepared by urea and by NaOH. after that, they converted to Ni/Fe LDH by olation reactions and then crystallization^60,61^. Meanwhile, the interaction between urea and (Ni and Fe) metal ions may be the reason of gradual morphology change from layers into the microspheres structure to decrease the surface energy^61^. Furthermore, this like morphology can expose the active site channel for the prepared adsorbate, which was beneficial to the improvement of the adsorption capacity. By addition of GL (H_2_O: GL 1:4.5), the plate shape like morphology decreased and only sphere-like samples were observed (Fig. 3(b)) due to the aggregation with smooth layer by-layer nanoplates structure. Slow precipitation can be occurred due to the interaction through complex formation of urea with metal ions causing gradual LDH formation which give a chance for glycerol molecules to form further coordination bonds with nickel and iron metal ions and these glycerol molecules form vesicles that act as a template for LDH so sphere like structure appear. Finally, both urea and GL have essential roles in the morphological conversion^58^. The EDX analysis of Ni/Fe LDH and Ni/Fe LDH/GL is demonstrated in Fig. 3(f,g). The corresponding HRTEM image of Ni/Fe LDH (Fig. 3(d)) reveals that there are plenty of thin nanoflakes aggregates on each other, this phenomenon related to the coprecipitation method of preparation^62^. The image of Ni/Fe LDH/GL was also given in Fig. 3(e) which also showed strong evidences of loading of GL on LDH surface.

#### Surface area and pore size distribution

**Figure 4:**
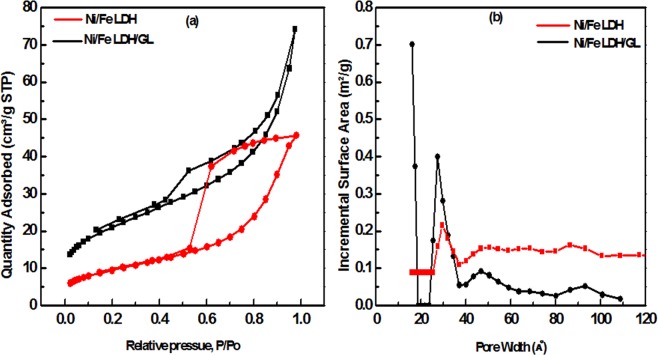
(**a**) N_2_ sorption isotherms and (**b**) Pore size distributions.

The surface area and porosity of the Ni/Fe LDH and Ni/Fe LDH/GL were studied using nitrogen adsorption/desorption isotherms. The measured BET surface area of Ni/Fe LDH/GL was 74.13 m^2^/g. While of Ni/Fe LDH was 34.21 m^2^/g. It can be seen that introducing glycerol to the Ni/Fe LDH result in an obvious increase in surface area and porosity. It is apparent from the adsorption and desorption curves (Fig. 4(a)) that Ni/Fe LDH exhibits a isotherm type IV and H2 hysteresis shape according IUPAC classification^63^, the desorption branch is likely to be dependent on network and/or ‘ink bottle’ effects^64^ which is suggested to be due to mesopores and capillary condensation^65^. After modification, in case of Ni/Fe LDH/GL, It was observed that there is a narrow adsorption–desorption hysteresis loop with relative pressure above 0.4, it turned to type-H3 hysteresis loop with slit shape pores ascribed to open end pores and ink-bottle pores^66^. Table 1 summarized the results of surface texture. In order to design a specific nanomaterial, controlling pore size, area and distribution will achieve our target^67^. Figure 4(b) present micro- and mesoporous distribution with the peak pore size of 1.6, 2.63 nm (Ni/Fe LDH/GL) and 2.52 nm (Ni/Fe LDH), respectively. The SSA of Ni/Fe LDH is attributed to the mesopores while the increase in the SSA of Ni/Fe LDH/GL resulted from accumulation of the recently developed micropores and the existing mesopores. As shown in Table 1, the high SSA of Ni/Fe LDH/GL attributed mainly to surface area of micropores and mespores while SSA of Ni/Fe LDH attributed only to surface area of mespores.

**Table 1:** Surface texture properties of Ni/Fe LDH and Ni/Fe LDH/GL.

Item	Ni/Fe LDH	Ni/Fe LDH/GL
BET Surface Area:	34.2181 m²/g	74.1317 m²/g
t-Plot micropore volume	0.0003 cm³/g	0.0016 cm³/g
BJH cumulative volume of pores	0.06 cm³/g	0.068 cm³/g
Average Pore Size (BJH method)	54.55 Å	41.932Å

Zeta potential of materials is an important property that explains many water treatment mechanisms. It is a measure of the magnitude of the charge repulsion/attraction between particles in solution. It is one of the main factors known to affect suspension stability, and provides details about the causes of dispersion or aggregation. The zeta potentials of aqueous dispersions of the Ni/Fe LDH and Ni/Fe LDH/GL were 43.3 and 32.8 mV, respectively, at pH 6 (Fig. S3(c,d)). The results show that the low positive zeta potential value of the Ni/Fe LDH/GL nanocomposite can be due to the interactions between the inorganic material and organic molecules (GL). This decrease is expected to be based on electrostatic considerations. The size distribution intensities of the Ni/Fe LDH and Ni/Fe LDH/GL were 270 and 469 nm, respectively as shown in Fig. S3(a,b).

### Adsorption study

#### Effect of adsorption pH

**Figure 5:**
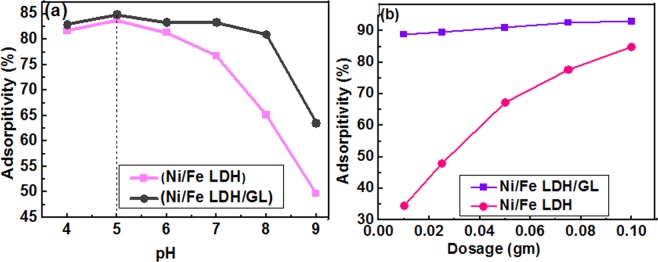
(**a**) The effect of pH on the removal efficiency of dichromate ions [20 ppm, 50 ml, and 0.025 gm catalyst], and (**b**) the effect of catalyst dose on the removal efficiency of dichromate ions [50 ppm, 50 ml, and pH = 5].

**Figure 6:**
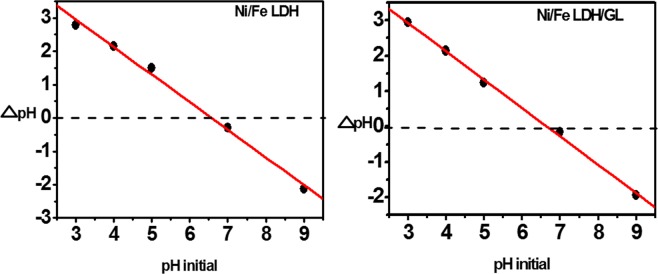
Represents pH_PZC_.

The optimum pH value for dichromate uptake from water can be obtained through studying the adsorption process over a range of pHs. The pH of the solution has a great effect on the adsorption process of the dichromate anions because an hexavalent chromium solution has different species at different pH values according to literature as chromate (CrO_4_^2−^), dichromate (Cr_2_O_7_^2−^), hydrogen chromate (HCrO_4_^−^), chromic acid (H_2_CrO_4_) and others^68^. Also, LDH characteristics and structure are greatly affected by pH value where, at lower pHs (<3) LDH disordering may be occurred^69^. As shown in Fig. 5(a), the effect of pH on adsorption process was studied over pH range from 4 to 9. The Cr(VI) uptake efficiency decreased with increasing of pH value and pH 5 was found to be the optimum value for adsorption of dichromate anions. This can be explained through studying the chemical properties of both chromium species and catalyst surface. At lower pH level the predominant chromium species are (Cr_2_O_7_^2−^) and (HCrO_4_^−^)^68^ and there is high concentration of H^+^ ions which neutralize the adsorbent surface and hence decrease the hindrance to chromium penetration^70^. At higher pH level the significant species is (CrO_4_^2−^) however, the absorption percentage decrease this may be due to high concentration of OH^−^ ions (basic medium) which compete with negatively charged Cr(VI) species for the active sites of the adsorbent surface^71^. The charges of the material surfaces and the ionization of the adsorbates can be greatly affected by the pH of the solution^72^ potassium dichromate has 2 values of pKa (strongest acidic pKa = −2.3) and (strongest basic pKa = −6.1) and this means that when pH values are lower or higher than pKa values of dichromate it becomes positively or negatively charged, respectively. The pH_PZC_ is the pH at which the material is neutral and has equal positive and negative charges^73^ and the material surface will be positive at pHs lower than the pH_PZC_ and negative at pHs higher than the pH_PZC_ here, in this study the pH_PZC_ was 6.8 (Fig. 6) so the LDH surfaces were occupied by positive charges at pHs less than 6.8. From the experimental data, as shown in Fig. 5(a), the maximum adsorption capacity of chromium was found at pH in the range from 5 to 7, which may be due to the electrostatic attraction between the opposite charges of dichromate and LDH materials. Adsorption of amount of chromium ions at pH higher than pH_PZC_ (LDH were negatively charged) indicate that adsorption not only due to electrostatic attraction effect but also may be due to hydrogen bond formation and ion exchange^74–76^.

#### Effect of adsorbent dose

The uptake of Cr(VI) increased with increasing Ni/Fe LDH and Ni/Fe LDH/GL concentration up to 0.1 gm and this may be accounted for increasing available active sits for adsorption (Fig. 5(b)). The absorptivity (%) in case of Ni/Fe LDH/GL was higher than that of Ni/Fe LDH where, at 0.01 gm of both catalysts the adsorption efficiency reached 88.87% and 34.44% and at 0.1 gm it reached 93% and 84.89% for Ni/Fe LDH/GL and Ni/Fe LDH, respectively and this may be related to introducing glycerol molecules during preparation which causes an obvious increase in surface area as shown in BET measurements, and also provide more active sites including oxygen-containing functional groups such as –OH, C–O, and C–O–H as demonstrated in FT-IR analysis so Cr (VI) ions can be removed by hydrogen bond formation and complexation with these oxygen-containing groups^77^.

#### Adsorption isotherm studies

**Figure 7:**
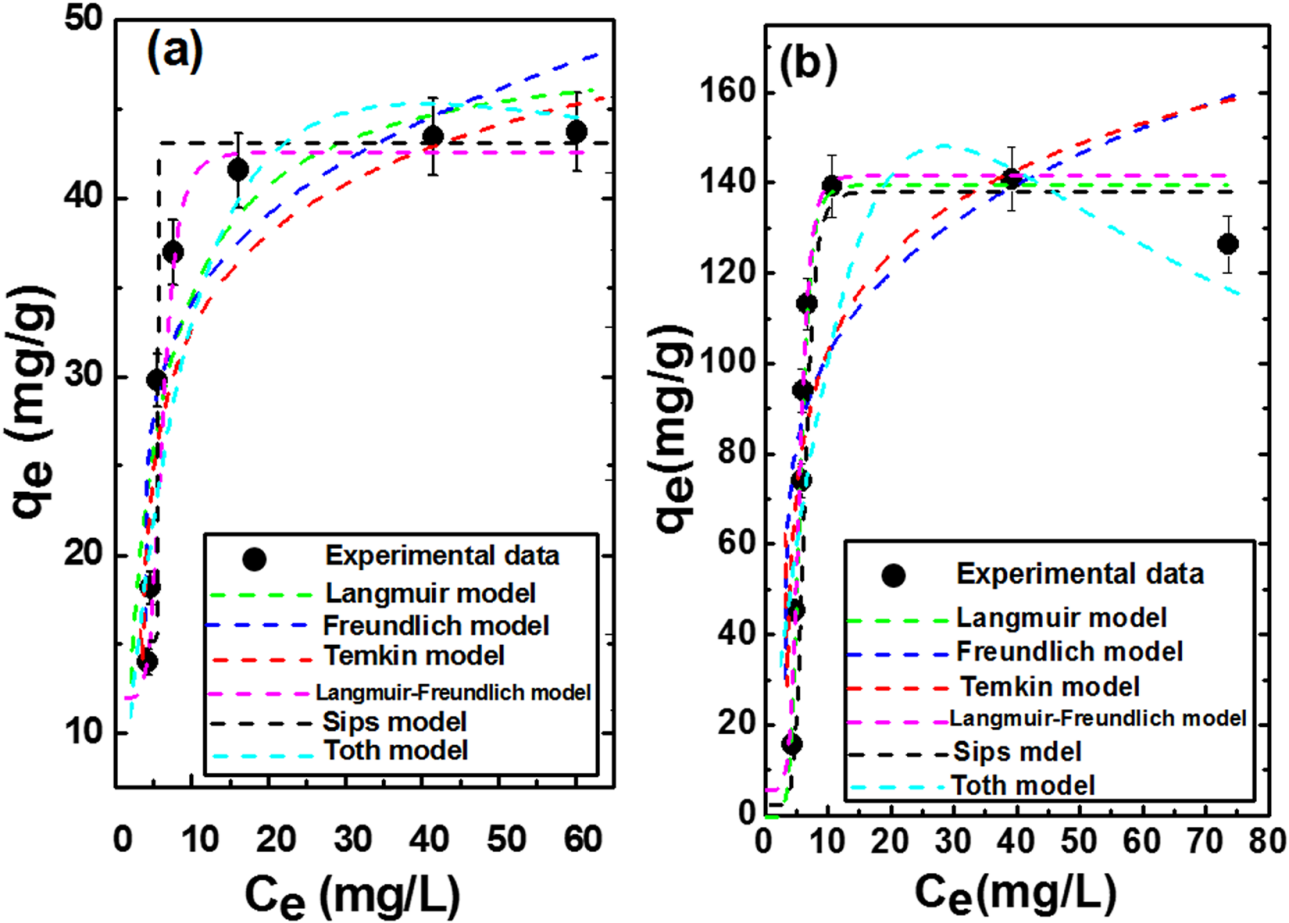
Adsorption isotherm models for (**a**) Ni/Fe LDH, and (**b**) Ni/Fe LDH/GL.

Adsorption isotherm represents the amount of adsorbate per unit mass of the catalyst as a function of its concentration at constant temperature and pH^78^. It can be used to calculate the maximum adsorption capacity of dichromate ions and describe how substances are adsorbed onto the surface of the catalyst^79^. as shown in Fig. 7, for both catalysts the amount of adsorbed dichromate ions increase by increasing its concentration because high initial dichromate concentration give a chance for each active site in the catalyst to be surrounded by more dichromate ions so more adsorption would be achieved, but the adsorbed amount in case of Ni/Fe LDH/GL is more than that of Ni/Fe LDH indicating that more active sites were provided by introducing glycerol during the preparation of LDH. In this study the most popular models (Langmuir, freundlich, Temkin, Sips, Toth and Langmuir-Freundlich) were be applied for the explanation of adsorption isotherms. For the two parameter models, the Langmuir model assumes that adsorption takes place at specific identical adsorption sites localized on the surface of the catalyst covering it with a monolayer of the adsorbate^3^. Langmuir adsorption isotherm is given by Eq. (1)

1 $${q}_{e}={q}_{{\max }}\frac{{K}_{L}{C}_{e}}{1+{K}_{L}{C}_{e}}$$

where, C_e_ (mg/l) is the concentration at equilibrium, q_e_ (mg/g) is the amount of the adsorbed molecules per unit mass of the catalyst, q_max_ (mg/g) is the maximum adsorption capacity and K_L_ is Langmuir constant related to the adsorption rate.

The separation factor R_L_ that determine the affinity between adsorbent and adsorbate can be calculated by applying Eq. (2)^80,81^.

2 $${R}_{L}=\frac{1}{1+{K}_{L}Co}$$

where, K_L_ (l/mg) is the Langmuir constant and C_o_ (mg/l) is the initial concentration of dichromate ions. by knowing R_L_ parameter value we can predict whether the shape of the isotherm is favorable (0 < R_L_ < 1), unfavorable (R_L_ > 1), linear (R_L_ = 1) or irreversible (R_L_ = 0)^82^. Here, in this study R_L_ is 0.097 and 0.144 for Ni/Fe LDH and Ni/Fe LDH/GL respectively, showing favorable isotherm for Ni/Fe LDH. From the experimental data the Langmuir maximum adsorption capacity of dichromate ions on Ni/Fe LDH was approximately (50.43 mg/g). The freundlich isotherm model describes a multilayer adsorption of adsorbed molecules onto heterogeneous adsorbent surface^15^. Freundlich isotherm can be represented by applying Eq. (3).

3 $${Q}_{e}={K}_{f}{{C}_{e}}^{1/n}$$

where, Q_e_ (mg/g) is the amount of adsorbed substances, C_e_ (mg/l) is the concentration at equilibrium, K_F_ (mg/g) is freundlich constant that gives information about adsorption capacity of adsorbed molecules onto catalyst surface^15^ and n is freundlich constant. 1/n give indication about adsorption intensity and surface heterogeneity^83^. 1/n usually < 1, if favorable adsorption^84^. Temkin model suggest uniform distribution of binding energies (up to maximum binding energy) and linearly decreasing of adsorption heat of particles in the layer regardless very low and very high concentrations^85^. Temkin adsorption isotherm is given by using Eqs. (4–7).

4 $${q}_{e}=\frac{RT}{{b}_{T}}\,\mathrm{ln}({A}_{T}{C}_{e})$$

5 $$qe=\frac{RT\,}{{b}_{T}}\,\mathrm{ln}\,{A}_{T}+\frac{RT}{bT}\,\mathrm{ln}\,{C}_{e}$$

6 $$B=RT/{b}_{T}$$

7 $${q}_{e}=B\,\mathrm{ln}\,{A}_{T}+B\,\mathrm{ln}\,{C}_{e}$$

where, A_T_ is Temkin isotherm equilibrium binding constant (l.g^−1^), b_T_ is Temkin isotherm constant, R is universal gas constant (8.314 J.mol^−1^.K^−1^), T is temperature at 298 K and B is constant related to heat of sorption (J.mol^−1^). Three parameter models (Sips, Langmuir-freundlich and Toth) were also applied. Sips isotherm model represents a mix of Langmuir and freundlich isotherm models. The parameter of this model are governed by experimental conditions (pH, temperature and concentration)^86^ at high concentration of adsorbate, it indicates a monolayer adsorption related to Langmuir isotherm while, at low concentration it reduces to freundlich isotherm^86^. By applying sips model, the maximum adsorption capacity of dichromate ions on Ni/Fe LDH/GL (135.857 mg/g) is approximately 4 times higher than that of Ni/Fe LDH (32.5114 mg/g). Sips model can be represented by Eq. (8).

8 $${q}_{e}=({q}_{max}{K}_{s}{C}_{e\,}^{1/n})(1+{K}_{s}{C}_{e}^{1/n})$$

where, q_max_ is the adsorption capacity (mg/g), K_s_ is sips constant related to adsorption energy and 1/n is sips isotherm exponent. Toth isotherm model is a modified form of Langmuir model and it is principally used for predicting the systems which cover both high and low adsorbate concentration^87,88^. Toth model can be presented by using Eq. (9)^87^.

9 $${q}_{e}=\frac{{K}_{e}{C}_{e}}{{[1+{({K}_{L}Ce)}^{n}]}^{1/n}}$$

where, K_l_ and n are Toth isotherm constants (mg/g) the parameter n give an indication of heterogeneity of the system where being more than unity the system is considered to be heterogeneous^88,89^. Langmuir-Freundlich isotherm model give an indication about heterogeneous surfaces and the distribution of adsorption energy on these surfaces^90^. At low concentration of adsorbate the system follows freundlich model while at high concentration of adsorbate it follows Langmuir model^91^. This model is expressed by Eq. (10).

10 $${q}_{e}=\frac{{q}_{MLF}{({K}_{LF}{C}_{e})}^{MLF}}{1+{({K}_{LF}{C}_{e})}^{MLF}}$$

where, q_MLF_ is Langmuir-Freundlich maximum adsorption capacity, K_LF_ is the equilibrium constant for heterogeneous solid and MLF is the heterogeneous parameter. The higher the value of MLF parameter, the larger is the degree of homogeneity^91^. From the correlation coefficients (R^2^) presented in Table 2, we can concluded that the adsorption data best fitted the Sips, Langmuir-Freundlish > toth > Temkin > Freundlish > Langmuir isotherm models, which meant that the adsorption process of Cr(VI) into Ni/Fe LDH/GL didn’t fit with Langmuir. The calculated maximum adsorption capacity from Sips was 135.86 mg/g, and according to Langmuir-Freundlish isotherm model was 136.05, the values of adsorption capacity calculated from Sips and Langmuir-Freundlish were very close values. The maximum adsorption capacity for the Cr(VI) using Ni/Fe LDH is calculated using Langmuir isotherm model was 50.43 mg/g.

**Table 2:** Showing all parameters of applied isotherm models.

Isotherm models	Parameters	Ni/Fe LDH	Ni/Fe LDH/GL
Langmuir	q_max_(mg/g)	50.43	188.65
	K_L_ (l/mg)	0.185	0.119
	R_L_	0.097	0.144
	R^2^	0.981	0.784
Freundlich	K_f_	16.81	50.29
	1/n	0.254	0.251
	N	2.63	2.71
	R^2^	0.969	0.902
Temkin	b_T_	260.546	52.47
	A_T_	2.5150	0.809
	R^2^	0.975	0.934
Sips	q_max_	32.5114	135.857
	K_s_	0.191	9.198
	1/n	30.827	6.790
	R^2^	0.913	0.994
Toth	K_e_	7.087	25.705
	K_l_	0.0331	0.0042
	n	1.281	1.760
	R^2^	0.984	0.956
Langmuir-Freundlich	q_MLF_	42.66	136.05
	K_LF_	0.206	0.181
	MLF	5.028	6.770
	R^2^	0.999	0.994

The parameters of all models are displayed in Table 2. Ni/Fe LDH/GL displays higher adsorption capacity toward dichromate ions than many other adsorbents (Table 3)^39,92–99^. These experimental results demonstrate that this modified LDH is an excellent adsorbent for dichromate uptake from aqueous solutions.

**Table 3:** Maximum adsorption capacity of some adsorbents for Cr(VI) ions removal.

Adsorbent	pH	Time (min)	Concentration of Cr (VI) (mg/l)	Adsorption capacity (mg/g)	Source
Aluminum –magnesium mixed hydroxide	2.5–5	90	200	105.3–112	^92^
LDHNSs	6	8	20–200	125.97	^93^
maghemite nanoparticles	2.5	15	50	19.2	^94^
Fe_3_O_4_@C@MgAl-LDH	6	60	100	152	^95^
flowerlike α-Fe_2_O_3_	3	180	25	30	^96^
Poly aniline-polyethylene glycol composite	5	30	50	68.97	^97^
*Melaleucadiosmifolia*leaf	7	120	250	62.5	^98^
Halloysite nanotubes	3	30	50	6.9	^99^
Zn/Al LDH	5.2	15	2.00	10.23	^39^
Zn/Al/Ala LDH	5.2	15	2.00	12.25	^39^
Ni/Fe LDH/GL	5	30	50	136.05	This study
Ni/Fe LDH	5	30	50	50.43	This study

#### Adsorption kinetics

**Figure 8:**
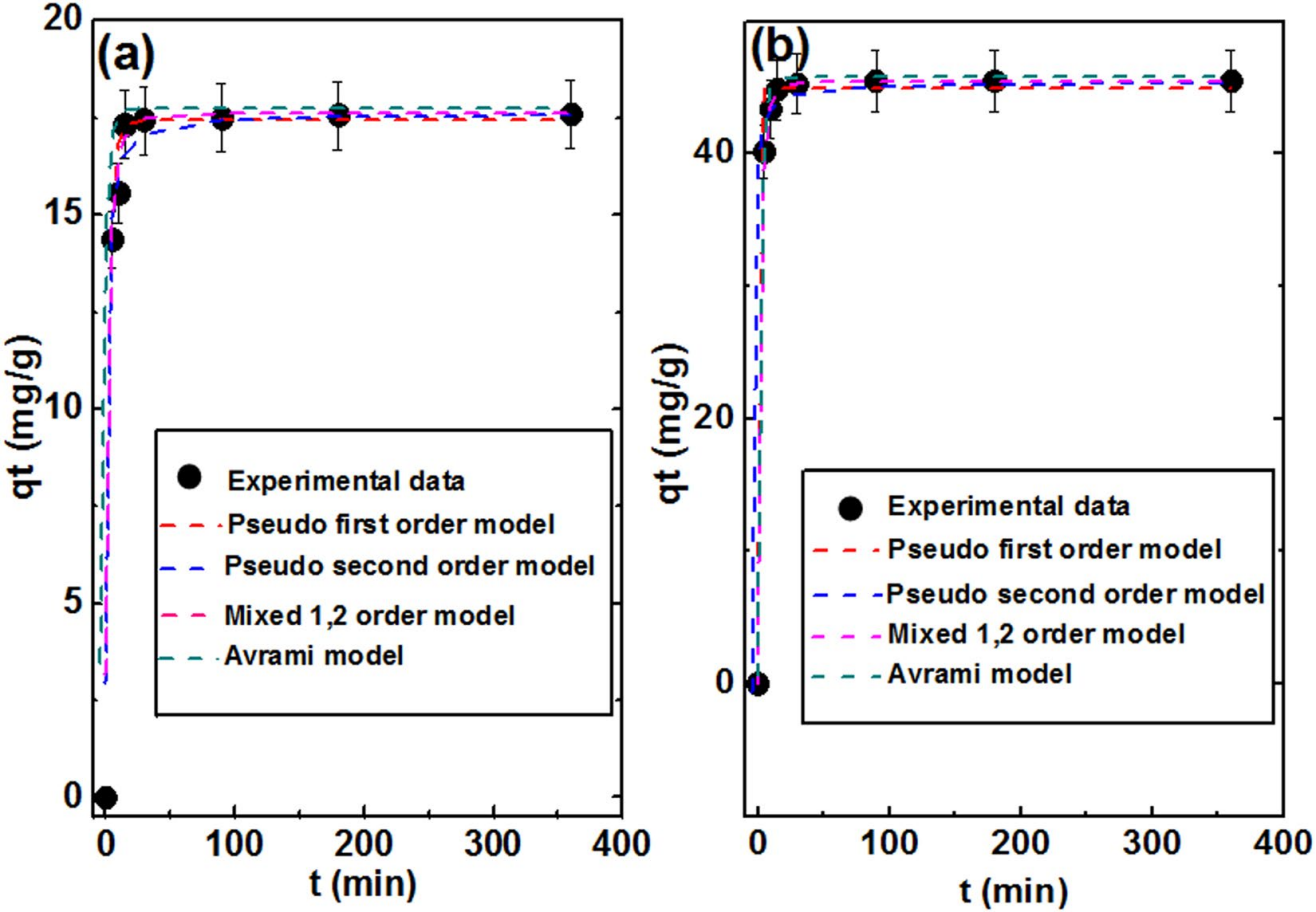
Adsorption kinetic models of (**a**) Ni/Fe LDH, and (**b**) Ni/Fe LDH/GL.

The effect of time on the removal efficiency was evaluated through applying pseudo first^100,101^ and second^102–104^ order, mixed 1,2 order^105^, intraparticle diffusion^106,107^ and Avrami^108–110^ models, as described by Eqs. (11–15), respectively. All parameters related to these kinetic models are reported in Table 4. From the experimental data demonstrated in Fig. 8, it is obvious that through the first 10 min there was a fast increasing in the up take efficiency which may be accounted for the more available vacant active sites then, it showed less and gradually adsorption behavior until 30 min after that, saturation and equilibrium is reached with no change in removal percent up to 360 min. for both Ni/Fe LDH and Ni/Fe LDH/GL, the adsorption process was best fitted with pseudo-first order, Avrami model, pseudo second order and 1,2 mixed order models as presented in R^2^ shown in Table 4 also it was modest fitted with intraparticle diffusion model with R^2^ (0.715 and 0.699), respectively.

11 $${q}_{t}={q}_{e}(1-{e}^{-{k}_{1}t})$$

12 $${q}_{t}=\frac{{{q}_{e}}^{2}{k}_{2}t}{1+{q}_{e}{k}_{2}t}$$

13 $${q}_{t}={q}_{e}\frac{1-\exp (\,-\,kt)}{1-{f}_{2}\exp (\,-\,kt)}$$

14 $${q}_{t}={k}_{ip}\sqrt{t}+{c}_{ip}$$

15 $${q}_{t}={q}_{e}[1-\exp {(-{k}_{av}t)}^{{n}_{av}}]$$

where, q_t_ is the adsorption capacity (mg g^−1^) at time t (min), q_e_ is the adsorption capacity (mg g^−1^) at equilibrium, k_1_ is the pseudo first order rate constant (min^−1^), k_2_ is the pseudo second order rate constant (min^−1^), f_2_ is the mixed 1,2 order coefficient (dimensionless), k is the adsorption rate constant (mg g^−1^ min^−1^), k_ip_ is the measure of diffusion coefficient (mg g^−1^ min^−1(1/2)^), c_ip_ is the interaparticle diffusion constant (mg g^−1^), k_av_ is the Avrami rate constant (min^−1^) and n_av_ is the Avrami component (dimensionless).

**Table 4:** Represent the kinetic models parameters.

Kinetic models	Parameters	Ni/Fe LDH	Ni/Fe LDH/GL
Pseudo first order	K_1_	0.324	87.136
	q_e_	17.36	44.29
	R^2^	0.999	0.998
Pseudo second order	K_2_	0.0481	0.033
	q_e_	17.850	45.97
	R^2^	0.999	0.999
Mixed 1,2 order	K	0.0988	0.1332
	q_e_	17.579	45.484
	f_2_	0.849	0.8737
	R^2^	0.999	0.999
Avrami	q_e_	17.367	45.145
	K_av_	1.645	1.372
	n_av_	0.1971	0.313
	R^2^	0.999	0.999
Intraparticle diffusion	K_ip_	1.095	2.819
	C_ip_	3.953	10.746
	R^2^	0.715	0.699

#### Thermodynamic study

**Figure 9:**
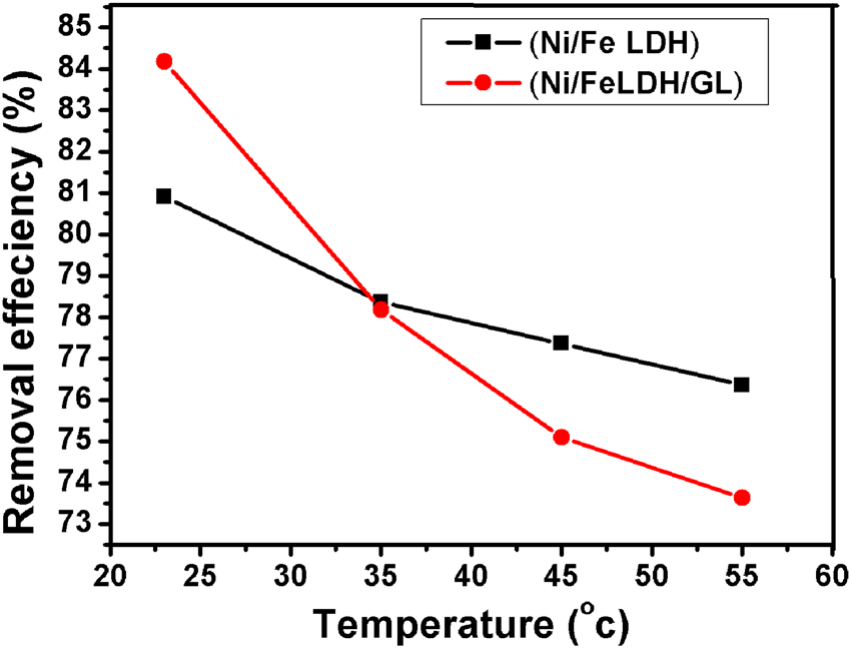
Effects of temperature on Cr(VI) removal efficiency using Ni/Fe LDH and Ni/Fe LDH/GL.

The effect of temperature on the Cr(VI) ions adsorption on Ni/Fe LDH and Ni/Fe LDH/GL was evaluated at 23 °C, 35 °C, 45 °C and 55 °C. Figure 9 shows the effect of temperature on the chromium ions removal efficiency. The experimental results and k_d_ (q_e_/c_e_) values at different temperature were employed to calculate the thermodynamic parameters (∆H°, ∆S° and ∆G°) by using Eq. (16) to predict the adsorption process nature^111,112^.

16 $$\mathrm{ln}\,{k}_{d}=\frac{\varDelta {S}^{^\circ }}{R}-\frac{\varDelta {H}^{^\circ }}{RT}$$

where, k_d_ is the equilibrium constant (L/mg), ∆H° is the enthalpy change of adsorption (kJ/mol), ∆S° is the entropy of adsorption (J/K mol), R is the gas rate constant (8.314 J/mol K) and T is the temperature in Kelvin (K). Enthalpy (∆H°) and entropy (∆S°) of adsorption could be calculated from the slope and the intercept of the straight line obtained by plotting lnk_d_ against 1/T and Gibbs free energy (∆G°) could be obtained using Eq. (17)

17 $$\varDelta {{\rm{G}}}^{^\circ }=-\,{\rm{RT}}\,{{\rm{lnk}}}_{{\rm{d}}}$$

The thermodynamic parameters of this system are reported in Table 5. Obtaining ∆H° in negative value predict an exothermic adsorption process so increasing the temperature of the system does not usually favor the process^15^. Entropy (∆S°) was reported in a negative value and this shows a decrease in randomness at the interface between solid and liquid in the adsorption of dichromate ions on LDH materials^113^. The negative values of Gibbs free energy (∆G°) show a spontaneous adsorption process. From the experimental data, increasing the temperature causes increasing in ∆G° values (decreasing in negativity) i.e. decreasing the degree of spontaneity and this may be related to the exothermic behavior of the reaction between Cr (VI) ions and both Ni/Fe LDH and Ni/Fe LDH/GL^114^.

**Table 5:** Thermodynamic parameters for adsorption of Cr (VI) ions on both Ni/Fe LDH and Ni/Fe LDH/GL.

	T (K)	∆G°(kJ/mol)	∆H°(kJ/mol)	∆S°(J/K mol)
Ni/Fe LDH	296	−1298.597	−6.6986	−18.415
	308	−949.700		
	318	−826.517		
	328	−698.632		
Ni/Fe LDH/GL	296	−4113.865	−16.2622	−41.57
	308	−3267.982		
	318	−2916.878		
	328	−2800.938		

#### Effect of coexisting anions and HA

**Figure 10:**
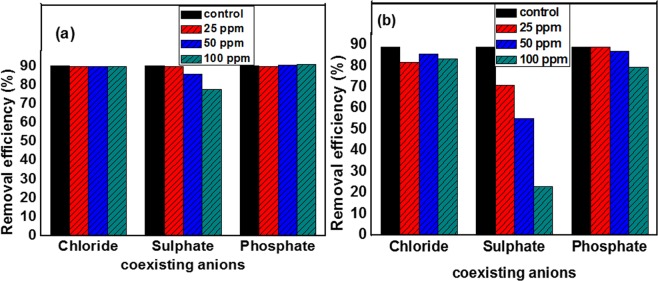
Effect of coexisting anions on removal capacity of dichromate ions onto (**a**) Ni/Fe LDH and (**b**) Ni/Fe LDH/GL.

Figure 10 represents the effect of interfering anions such as Cl^−^, SO_4_^2−^, and HPO_4_^2−^ with various concentrations of 25, 50 and 100 ppm on the adsorpitivity of dichromate ions. As shown, increasing the concentration of chloride anions caused no significant difference in the adsorpitivity of Cr (VI) ions onto Ni/Fe LDH while, it caused a great decreasing in removal efficiency in case of Ni/Fe LDH/GL. This may be related to competitive behavior between chloride anions and dichromate to be adsorbed onto the active sites of LDH materials. sulphate anions had a more pronounced impact on the adsorption capacity where it made the adsorpitivity of dichromate ions to be lowered from 90.48% to 78.06% and from 88.88% to 22.9% onto Ni/Fe LDH and Ni/Fe LDH/Gl, respectively when it was added with concentration of 100 ppm and this impact can be attributed to greatly competitive effect of high charged anions through electrostatic attraction to the catalyst^115^. Also, difference between the anions effect on the adsorption efficiency may related to the value of Z/r (charge/ radius) for SO_4_^2−^ is bigger, the tendency of SO_4_^2−^ uptake on active sites of used adsorbents is higher and less Cr(VI) adsorption. On the other hand, in basic environment, at optimum pH phosphate anion will be transformed to HPO_4_^2−^ and PO_4_^3−^ ions which being have a charge density higher than nitrate anions resulting in a lower adsorption efficiency for Cr(VI). The same results are reported in previous studies^116^.

**Figure 11:**
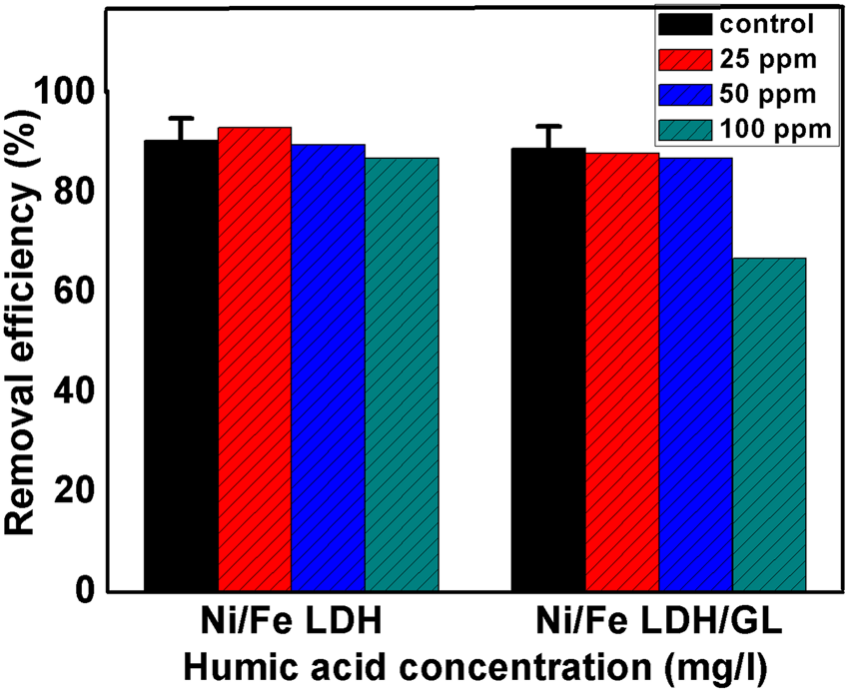
Effect of humic acid on adsorpitivity of Cr (VI) ions onto Ni/Fe LDH and Ni/Fe LDH/GL.

Organic humic acid can be found together with Cr (VI) ions in wastewater. As shown in Fig. 11 increasing the concentration of humic acid in the solutions negatively affected the adsorption capacity of dichromate ions and this trend can be accounted for two reasons: firstly, the available reactive sites of LDH materials were consumed by humic acid molecules i.e. HA competed with dichromate ions to be adsorbed onto LDH material. Secondly, the adsorbed humic acid particles promoted the electrostatic repulsion and hence prevented the mass transfer of negatively charged dichromate ions^117^.

#### Adsorbents chemical stability

**Figure 12:**
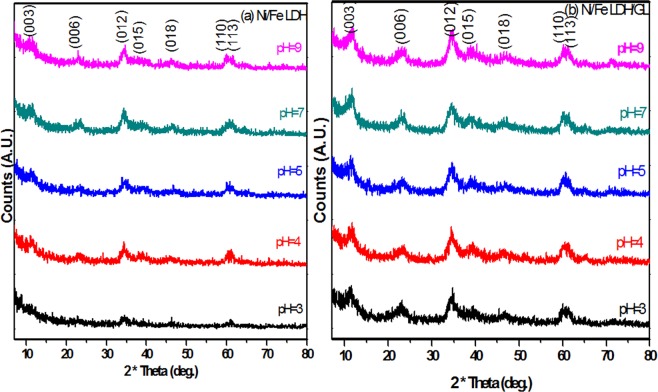
XRD patterns of (**a**) Ni/Fe LDH, and (**b**) Ni/Fe LDH/GL at different pHs.

The chemical stability of the prepared LDH materials was tested by analyzing XRD patterns of these adsorbents at different pHs (3–9). For Ni/Fe LDH (Fig. 12(a)) the intensities of bands were greatly weaker than that shown in Fig. 1(a) especially that at pH = 3 which possessed no crystal structure. However, for Ni/Fe LDH/GL nanocomposite (Fig. 12(b)), all band reflections were not affected at different pHs showing the same band intensities compared to XRD patterns Fig. 1(b) and exhibited intact layer-by-layer ordered structures even at low pHs. Finally, the addition of glycerol to LDH adsorbents obviously improved their stability.

#### Recyclability of the Nano-adsorbents

**Figure 13:**
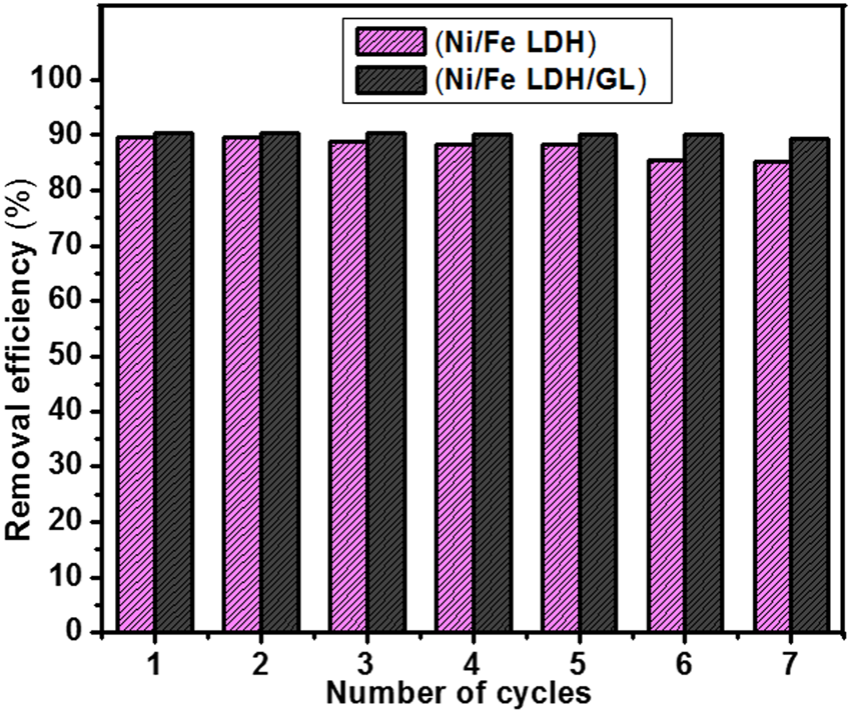
The reusability performance of Ni/Fe LDH and Ni/Fe LDH/GL.

It is of great important to determine the reusability performance of the exhausted adsorbents to evaluate their economic situation. As shown in Fig. 13, after 7 cycles of regeneration, the adsorption capacity was not greatly changed where, Ni/Fe LDH maintain about 88.2% of its removal efficiency after 5 cycles and about 85.2% after 7 cycles while, Ni/Fe LDH/GL maintain about 90% of its removal capacity after 5 cycles and about 89.3% after 7 cycles and this means that these materials have a good regeneration behavior.

#### Adsorption mechanisms

From FT-IR analysis after adsorption (Fig. 2) one can conclude the mechanisms of adsorption of dichromate anions onto LDH materials. The O-H absorption bands of Ni/Fe LDH and Ni/Fe LDH/GL were shifted from 3398 to 3387 cm^−1^ and from 3420 to 3408 cm^−1^, respectively after adsorption of Cr(VI) ions indicating the formation of hydrogen bonds between OH groups on the surface of LDHs and the oxygen of dichromate ions^56^ so Cr (VI) ions can be removed by complexation with hydroxyl groups. the metal oxide band at 787 cm^−1^ was shifted to 777 cm^−1^ in Ni/Fe LDH and disappeared in Ni/Fe LDH/GL because of the electrostatic attraction between negatively charged dichromate ions and higher valence metal ions (Ni^+2^, Fe^+3^)^56,118^ and this can be confirmed by zeta potential measurements. the intensity of nitrate absorption bands located at 1357 in Ni/Fe LDH and Ni/Fe LDH/GL after adsorption were decreased and this is attributed to the intercalated of dichromate anions by the ion exchange of NO_3_^−^ groups^119^ The results suggested that Cr (VI) ions were absorbed onto LDH through electrostatic attraction, ion exchange, complexation and hydrogen bond formation.

### Monte Carlo simulation

**Figure 14:**
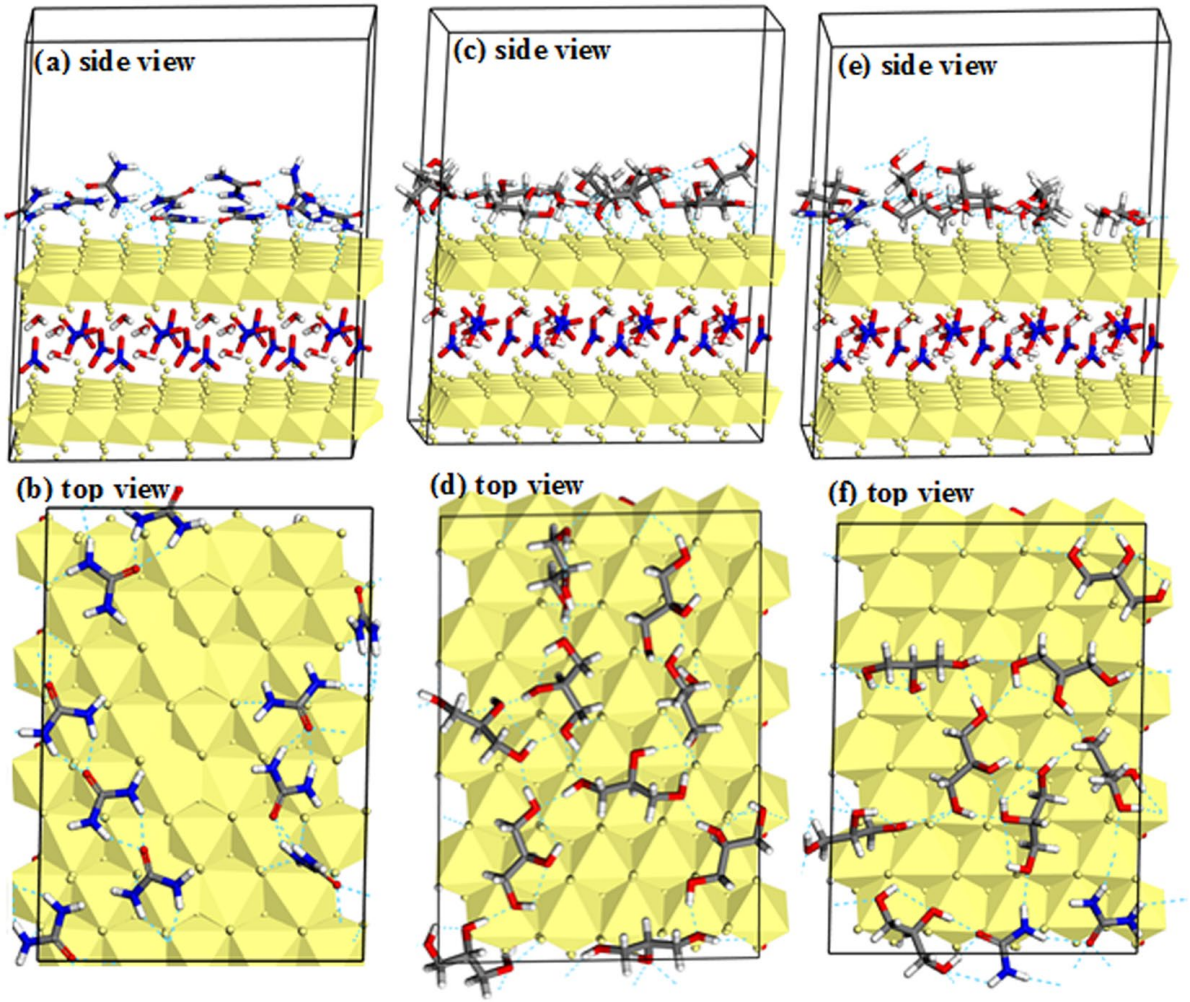
The lowest-energy structures obtained from the Monte Carlo simulations for (**a,b**) 10 molecules of urea + LDH system; (**b,c**) 10 molecules of glycerol molecules + LDH system; and (**e,f**) 8 molecules of glycerol + 2 molecules of urea + LDH system.

Figure 14(a–f) displays the interactions of urea and glycerol molecules with the Ni-Fe LDH surface. It can be seen form Fig. 14(a,b) that the nitrogen or oxygen atoms of urea molecules formed intermolecular hydrogen bonds with the hydroxyl hydrogen atoms of the LDH surface. A net of hydrogen bonds among the urea molecules were formed, in which each carbonyl oxygen atom formed two hydrogen bonds with both (-NH_2_) groups. Similarly, the hydroxyl oxygen atoms of glycerol molecules formed hydrogen bonds with LDH, Fig. 14(b,c). Intermolecular hydrogen bonds were formed among glycerol hydroxyl groups. Additionally, intramolecular hydrogen bonds were formed between the glycerol hydroxyl groups. Figure 14(e,f) shows the interactions of 4:1 (glycerol: urea) molar ratio with Ni-Fe LDH surface. Beside the hydrogen bonds between glycerol or urea with LDH surface, intermolecular hydrogen bonds were also formed between urea and glycerol molecules.

**Figure 15:**
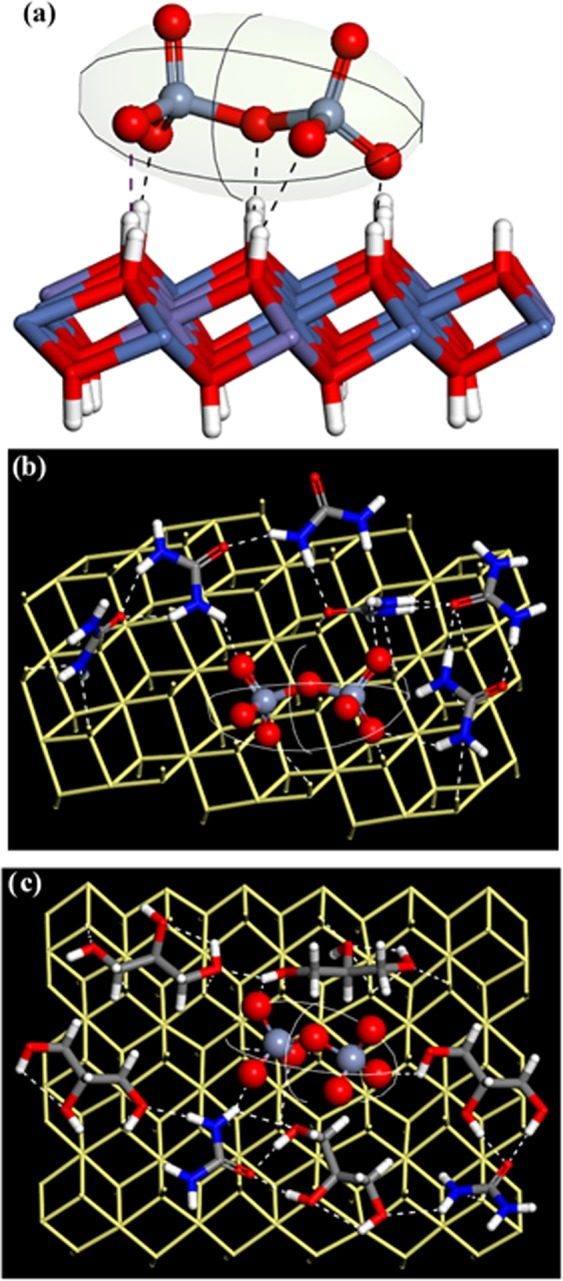
The MC lowest-energy configurations of the adsorption of a dichromate ion on (**a**) LDH, (**b**) 10 molecules of urea + LDH system, and (**c**) 8 molecules of glycerol + 2 molecules of urea + LDH system. For clarity purpose, we do not show the two layers and other molecules.

The adsorption of a dichromate ion on the LDH, urea-LDH, and 4:1 (glycerol/ urea)-LDH surfaces are shown in Fig. 15(a–c). The dichromate oxygen atoms were found to form five hydrogen bonds with the hydroxyl hydrogen atoms of LDH (Fig. 15a). In case of dichromate adsorption on urea-LDH, the dichromate oxygen atoms formed three hydrogen bonds with the LDH and another three hydrogen bonds with urea hydrogen atoms (Fig. 15b). While in case of dichromate adsorption on glycerol-urea LDH, the dichromate ion formed three hydrogen bonds with the LDH surface, two hydrogen bonds with two glycerol molecules, and a hydrogen bond with urea. Form the-above discussion, one can conclude that the presence of urea/glycerol molecules on the LDH surface enhances the dichromate adsorption.

## Experimental Section

### Materials

All used chemicals (Table S1) were of analytical grade and used without previous purification and bidistilled water was used during all experiments.

### Synthesis

#### Ni/Fe LDH

Ni/Fe LDH with molar ratio of 2:1 was synthesized by the simplest co-precipitation method. Nickel nitrate and ferric nitrate (molar ratio of 2:1) were dissolved in 50 ml H_2_O and urea was dissolved in 50 ml H_2_O forming a solution of 1.5 M. urea solution was added dropwise on metal ions solution under vigorous stirring at 40 °C then, a solution of NH_4_OH (2 M) was added dropwise with very slow rate until pH reached 8. After 24 h stirring, the prepared LDH was collected and washed many times using bi-distilled water and ethanol until pH 7 and then dried at 50 °C overnight.

#### Ni/Fe LDH/GL

Ni/Fe LDH/GL with molar ratio of 2:1 was synthesized by the same co-precipitation method. A 100 ml mixed solvent of glycerol and H_2_O with volume ratio of (4.5:1) was stirred for 10 minute at room temperature to form homogeneous solution. Nickel nitrate and ferric nitrate (molar ratio of 2:1) were dissolved in 50 ml of this mixed solvent and urea was dissolved in the 50 ml remain to form urea solution of 1.5 M. urea solution was added dropwise on metal ions solution under vigorous stirring at 40 °C then, a solution of NH_4_OH (2 M) was added dropwise with very slow rate until pH reached 8. After 24 h stirring, the prepared LDH was collected and washed many times using bidistilled water and ethanol until pH 7 and then dried at 50 °C overnight.

### Characterization

The as synthesized LDH materials were characterized by using different analysis techniques. X-ray diffraction (XRD) patterns were obtained with a PANalytical (Empyrean) X-ray diffraction using Cu K α radiation (wave length 0.154 cm^−1^) at an accelerating voltage 40 KV, current of 35 mA, scan angle range from 20° to 70° and scan step 0:02°. The Fourier transform infrared (FT-IR) spectra (4000–400 cm^−1^) were obtained by a Bruker (Vertex 70) spectrometer. Field emission scanning electron microscopy (FESEM) images were taken by (Gemini, Zeiss-Ultra55) field emission high resolution scanning electron microscope) and EDX for elemental analysis of materials. High resolution transmission electron microscopy (HRTEM) images were taken by JEOL-JEM 2100 (Japan) with an acceleration voltage of 200 KV. The BET (Brunauer, Emmett, and Teller) surface area of the as prepared materials was calculated from adsorption isotherms using a Quantachrome NOVA Automated Gas Sorption System. Zeta potential and partial size measurements were performed on a Malvern instrument (Malvern Instruments Ltd)^83^.

### Adsorption Experiments

The batch adsorption experiments were performed in 250 ml conical flasks through adding Ni/Fe LDH/GL and Ni/Fe LDH to Cr(VI) ions solutions and adjusting pH by pH meter (A∂wa–AD1030) through using 0.1 M NaOH and 0.1 M HCl. an orbital shaker (orbital shaker, SK-0330-pro) conducted at 200 rpm was used for shaking the prepared solutions at room temperature for 24 hr. To determine the concentration of Cr(VI) ions after adsorption; UV-visible spectrophotometer (Shimadzu UV-3600, Japan) was used at 350 nm^120^ after filtration the adsorbent by 0.22 µm pore size syringe filter (Millipore Millex-G, Hydrophilic PVDF). To compare the adsorption behavior of Cr(VI) ions between Ni/Fe LDH and Ni/Fe LDH/GL; many factors were studied such as pH ranging from 4 to 9, catalyst dose (0.20–2 g/l), dichromate ions concentrations ranging from 20 to 200 ppm, contact time from 5 min to 6 hr and finally temperature (23, 35, 45, 55 °C). The adsorptivity percent of Cr(VI) ions on Ni/Fe LDH and modified Ni/Fe LDH was calculated through Eq. (18).

18 $$Q \% =\frac{{C}_{o}-{C}_{t}}{{C}_{o}}\,\times \,100$$

where Q is adsorptivity percent, C_O_ is Cr(VI) ions initial concentration and C_t_ represents Cr(VI) ions concentration after adsorption at time t. equilibrium adsorption capacity was obtained by applying Eq. (19).

19 $${q}_{e}=({C}_{o}-{C}_{e})\,\frac{m}{v}$$

where, q_e_ (mg/g) represents equilibrium adsorption capacity, C_e_ is equilibrium concentration of Cr(VI) ions in mg/l, v is the volume of solution in litre and m is the mass of catalysts in grams. All experiments were performed in triplicate and the average values were calculated.

Point of zero charge of the Ni/Fe LDH and Ni/Fe LDH/GL were evaluated through adding 0.05 g from the prepared LDH materials to 25 ml NaCl (0.01 M) in 50 ml conical flasks and adjusting pH of the solutions at (3.0, 4.0, 5.0, 7.0, and 9.0). Then, the solutions were set for 24 h to determine the final pH. ∆pH (pH_final_ − pH_initial_) was plotted against the values of initial pH. PZC is equal to the initial pH at which the ∆pH = 0^15,121^.

To evaluate the influence of coexisting anions (Cl^−^, SO_4_^2−^ and HPO_4_^2−^) and humic acid on the removal capacity of Cr(VI) ions, 25 ml of 50 mg/l of Cr(VI) solutions containing different concentrations (25, 50 and 100 ppm) of interfering anions (as sodium salts) and humic acid with 0.0625 g of Ni/Fe LDH and 0.025 g of Ni/Fe LDH/GL were shaken at pH equal 5 for 24 h. the LDH materials were filtered and the concentrations of Cr(VI) ions in solutions were determined.

The chemical stability of LDH materials was carried out by adding 0.05 g of adsorbent into 50 ml of H_2_O and pHs were adjusted at (3.0, 4.0, 5.0, 7.0, and 9.0) using 0.1 M HCl and 0.1 M NaOH and were set for 1 week. Then, XRD analysis was performed on the tested LDH materials after drying them at 50 °C to examine their chemical stability^122^.

The recycling experiments are significant important factor in the suitability of prepared adsorbent for using and reduce the overall cost of treatment process. Adsorption desorption behaviour of Cr(VI) from Ni/Fe LDH and Ni/Fe LDH/GL was conducted using 0.05 N NaOH solution^95^ and stirred for 1 hr then, the concentration of Cr(VI) was determined by UV-visible spectrophotometer and LDH materials were washed by distilled water until pH reached 7 and dried at 50 ^o^C for 2 hr then, they were injected into another adsorption cycle to determine their regeneration efficiency.

### Computation details

Two layers of [Ni_12_Fe_6_(OH)_36_]6NO_3_∙6H_2_O were constructed as suggested by Fan *et al*.^123^. A vacuum slab with 15 Å was build above the two layers. This surface was optimized by density functional theory (DFT) with DNP basis set and GGA/RPBE functional. The core electrons of the Ni-Fe LDH were treated by the effective core potential. The DMol^3^ code^124^ was used in the DFT calculations. A large LDH surface, a (4 × 1 × 1) times of the optimized LDH, was built to be used in the Monte Carlo (MC) simulation. The Adsorption Locator module in BIOVIA Materials Studio (BIOVIA, 2017) package was used to carry out the MC simulation, which identifies the lowest-energy configurations. We have simulated six systems: (1) 10 molecules of urea + LDH; (2) 10 molecules of glycerol molecules + LDH; (3) 8 molecules of glycerol + 2 molecules of urea + LDH; (4) dichromate ion + LDH; (5) dichromate ion + system (1); and (6) dichromate ion + system (3). The forcefield COMPASS II^125^ was used in the MC simulation. The summation method was Ewald and group based for the electrostatic and van der Waals forces, respectively. The quality of the MC simulation was set to fine.

## Conclusion

Briefly, Ni/Fe LDH and modified Ni/Fe LDH/GL nanocomposite with spherical – like shape were synthesized by co precipitation method and were used for the removal of potassium dichromate from aqueous solutions by applying batch adsorption experiments and the results indicated that the adsorption process is strongly pH dependent with optimum pH = 5 and the removal precent increase by increasing the concentration of Cr(VI). The maximum adsorption capacity of Cr(VI) on Ni/Fe LDH/GL (136.05 mg/g) was higher than that on Ni/Fe LDH (50.43 mg/g). In addition, the adsorption processes on Ni/Fe LDH and Ni/Fe LDH/GL were simulated by pseudo-first order, Avrami model, pseudo second order and 1,2 mixed order models and they were spontaneous exothermic adsorption process. The investigation of coexisting anions and humic acid effect on the adsorption process of dichromate ions showed generally negative influence on the removal capacity. The modification of LDH materials by using glycerol improved their chemical stability at different pHs. these materials have a good regeneration behaviour even after exposed them to 7 cycles. The results suggested that Cr (VI) ions were absorbed onto LDH through electrostatic attraction, ion exchange, complexation and hydrogen bond formation. Finally, the fabricated materials are highly efficient adsorbents for removal of Cr(VI) from natural environments.

## Supplementary information

Supplementary Information.
