# Supplementary material for: Novel synthesis of Ni/Fe layered double hydroxides using urea and glycerol and their enhanced adsorption behavior for Cr(VI) removal
Source: Sci Rep. 2020 Jan 17;10:587. doi: 10.1038/s41598-020-57519-4 (PMC6969103; doi:10.1038/s41598-020-57519-4)
Supplement: Supplementary file 1 — Supplementary Information. [file 41598_2020_57519_MOESM1_ESM.docx]

**Supplemental information**

**Table S1**. The chemicals used in this work

| **IUPAC systematic name** | **Source of the chemicals** | **CAS registry numbers** | **Purity %** |
| --- | --- | --- | --- |
| Nickel nitrate hexahydrate | Alpha Chemika, India | 13478-00-7 | 99 |
| Iron nitrate nonahydrate | Alpha Chemika, India | 7782-61-8 | 98 |
| Urea | Piochem for laboratory chemicals, Egypt | 604389 | **-** |
| Glycerol | Sigma-Aldrich | 56-81-5 | 99.5 |
| Ethyl Alcohol | Diachem Chemicals, Egypt | 64-19-5 | 99.9 |
| Sodium hydroxide | Piochem for laboratory chemicals, Egypt | 1310-73-2 | 95 |
| Hydrochloric acid | Carlo Erba Reagent, Val de Reuil, near Paris (France) | 7647-01-0 | 37 |
| Potassium dichromate | Sigma-Aldrich | 7778-50-9 | 99 |
| Sodium chloride | Techno Pharmchem, Bahadurarh | 7647-14-5 | 99 |
| Sodium sulphate anhydrous | Research lab fin chem industries | 7757-82-6 | 99 |
| Disodium hydrogen phosphate anhydrous | Piochem for laboratory chemicals, Egypt | 10039-32-4 | 99 |
| Humic acid | Commercial humic acid | **-** | **-** |

**Fig. S1.** XRD of Ni/Fe LDH prepared by urea and NaOH

**Fig. S2.** FTIR of Ni/Fe LDHs prepared by urea and by NaOH


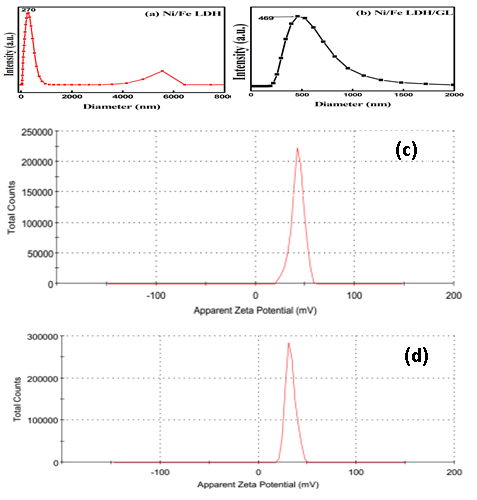


**Fig. S3.** Particle size of (a) Ni/Fe LDH and (b) Ni/Fe LDH/GL and zeta potential distribution of (c) Ni/Fe LDH and (d) Ni/Fe LDH/GL.
